# Secondary Dopants of Electrically Conducting Polyanilines

**DOI:** 10.3390/polym13172904

**Published:** 2021-08-28

**Authors:** Young-Gi Kim, Hai-Long Nguyen, Patrick Kinlen

**Affiliations:** 1 Department of Chemistry, Delaware State University, Dover, DE 19901, USA; 2 US Army Future Command, Picatinny Arsenal, Wharton, NJ 07806, USA; hai-long.v.nguyen.civ@mail.mil; 3 Department of Chemistry, University of Missouri, Columbia, MO 65211, USA; kinlenp@missouri.edu

**Keywords:** electrically conducting polymers, polyaniline, secondary dopants

## Abstract

Secondary dopants and the doping methods were identified for increasing the electrical conductivity of a highly processable and a primarily doped polyaniline dinonylnaphthalene sulfonic acid (PANI-DNNSA). The secondary doping was carried out using film, solution, and vapor doping methods. The doping methods and functional groups of secondary dopants were observed to play a critical role for inducing electrical characteristics of polyaniline. When secondary film doping method and *p*-toluenesulfonic acid were used, the electrical conductivity of the secondary doped polyaniline was measured to be increased from 0.16 to 334 S/cm. A novel vapor annealing doping method was developed to incorporate secondary dopants into solution cast polyaniline films.

## 1. Introduction

Conductive polymers (CPs) that have a broad range of electrical conductivity and electrochemical activity have attracted considerable interest due to their broad applicability in opto-electronic devices including polymer solar cells (PSC), field effect transistors (FETs), lasers, light emitting diodes (LEDs), electrochromic devices (ECDs), non-volatile memories (NVMs), fuel cells, batteries, and supercapacitors (SCs). Among these applications, CP- based SCs in which the CP can be used as an electrode are promising in view of having high specific capacitance and fast electron transfer characteristics while being light weight and flexible. Another application, PSC used to implement the CPs in hole transporting layer (HTL) and photoactive layer (PAL) successfully [1,2,3,4,5,6].

Past efforts to utilize CPs in opto-electronic applications were encumbered by the intractability and insolubility of the CP and required tedious electrochemical polymerization of monomers to form a conductive film. Polyaniline, on the other hand, can be synthesized to create a processible and soluble conductive polymer with controlled electrical conductivity governed by the nature of dopants employed [7]. Polyaniline films cast from solution may then be doped with selected solvents such as *m*-cresol and N-methyl-2-pyrrolidone to provide desirable electrical and optical properties in an easy way [8,9,10,11,12,13,14,15]. Polyaniline is the preferred CP for CP-SCs and has been shown to have excellent electrochemical capacity in electrochemical cells [16,17,18,19,20].

**Figure 1:**
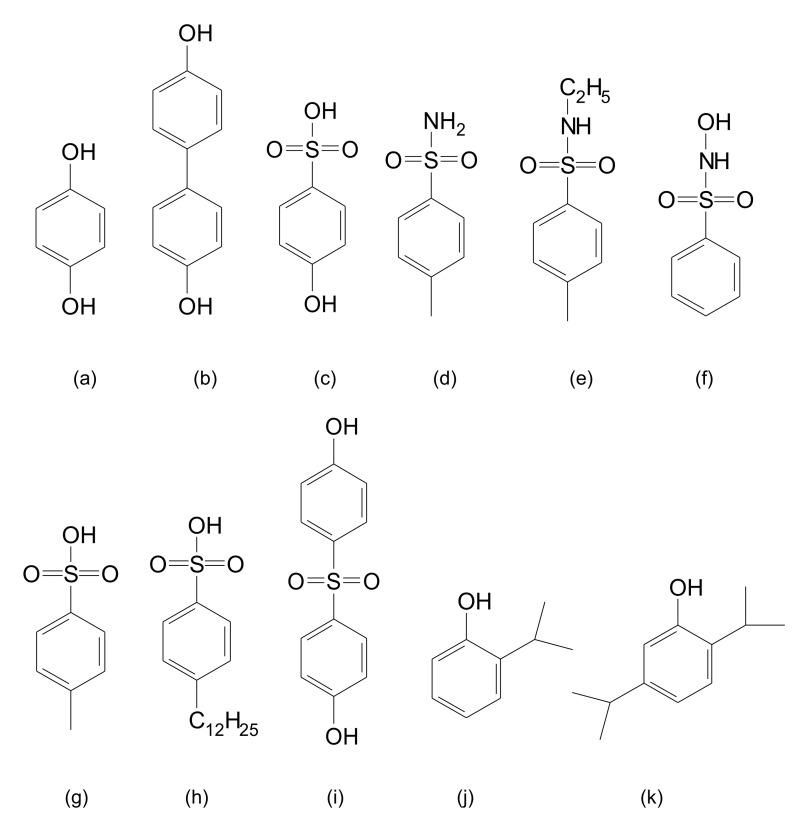
Chemical structures of secondary dopants: (**a**) HQ, (**b**) BP, (**c**) HBSA, (**d**) TSAm, (**e**) E-TSAm, (**f**) HBSAm, (**g**) PTSA, (**h**) DBSA, (**i**) SDP, (**j**) Thymol, and (**k**) Carvacrol.

Other than DBSA or DNNSA doped polyaniline, most “soluble” polyanilines reported in the literature have limited solubility (<5% *w/w*) or processability in organic solvents in the doped form and form precipitates or insoluble gels over time. PANI-DNNSA, on the other hand, is fully soluble in aromatic hydrocarbons up to 50% *w/w*. Solutions of PANI-DNNSA may be spin coated, drop cast, spray coated, dip coated, or screen printed to yield particle free coatings. This novelty in the processability of the primary doped polyaniline solution, PANI-DNNSA, allows for exploration of variable secondary doping methods that are not possible with other conductive polymers and provides a platform for creating conductive polymer coatings and formulations with near metallic conductivity. The primarily doped polyaniline showed a highly bulky structure in the SEM image which was formed by a long alkyl group-bearing primary dopant, DNNSA. In this paper, we report the identification of the novel secondary dopants for enhancing the electrical conductivity of a highly processable and a primarily doped polyaniline. In addition, the optical properties of the secondary doped polyaniline film are discussed as means of identifying the secondary dopants. The electrical conductivity (334 S/cm) reported in this article is one of the highest observed for a polyaniline film doped with *p*-toluenesulfonic acid as a secondary dopant. Figure 1 shows some examples of chemical dopants used in this report.

## 2. Materials and Methods

### 2.1. Materials

Polyaniline-dinonylnaphthalene sulfonic acid (PAC 1003, Crosslink, commercial grade is available from Boron Molecular, Noble Park, Victoria, Australia), hydroquinone (HQ, Sigma-Aldrich, St. Louis, MO, USA), thymol (Sigma-Aldrich), carvacrol (Sigma-Aldrich), *N*-ethyl-toluenesulfonamide (E-TSAm, Sigma-Aldrich), *N*-hydroxy-benzenesulfonamide (HBSAm, Fluka, Buchs, Switzerland), 4,4′-biphenol (BP, Sigma-Aldrich), 4,4′-sulfonyldiphenol (SDP, Acros, Hampton, NH, USA), hydroxybenzenesulfonic acid (HBSA, Sigma-Aldrich), *p*-toluenesulfonic acid (PTSA, J.T. Baker, Phillipsburg, NJ, USA), p-toluenesulfonamide (TSAm, Acros), 2-isopropylphenol (IPA, Sigma-Aldrich), 2,6-diisopropylphenol (Sigma-Aldrich), sulfuric acid (Sigma-Aldrich), acetone (Sigma-Aldrich), butyl cellosolve (Sigma-Aldrich), and xylene (Sigma-Aldrich) were a reagent grade and were used as received.

### 2.2. Methods

A 0.3-mL solution (15% conc.) of polyaniline dinonylnaphthalene sulfonic acid (PANI-DNNSA) was spin coated on a pre-cleaned glass slide (1″ × 1″) using a spin-coater (Brewer Science, Rolla, MO, USA) at speeds of 1000–8000 rpm for 30 s. After air drying in a fume hood for 30 min, the polyaniline film was dried in a vacuum oven (VWR International, Bridgeport, NJ, USA) at 150 °C for 30 min. The PANI-DNNSA film was treated with various secondary dopants in a 25–50 mL beaker using a dip doping (film doping), in situ doping (solution doping), or vapor doping method. After dipping film samples in secondary doping solution (5% *w/v*) for 30 s, the films were air dried in a fume hood. The air-dried films were thermally treated at 150 °C for 30 min.

In situ doping (solution doping) was carried out by mixing secondary dopants and PANI-DNNSA solution at a molar ratio of 0.3:1 if not specified. The in situ doped solution was applied in a spin coater and the coated sample was air dried in a fume hood. The air-dried films were thermally treated at 150 °C for 30 min.

Vapor doping was conducted in a 10 mL beaker containing 2 g of secondary dopant. These conditions insured that the vaporized dopant filled the beaker volume for complete exposure of the polyaniline film. The beaker was covered with a glass slide and gently heated on a hot plate to vaporize the secondary dopant. The polyaniline films were characterized using AFM (Bruker, Billerica, MA, USA), 4-probe I-V measurement (Keithley Instruments, Solon, OH, USA) (resistivity, surface resistant and conductivity), and UV–Vis–NIR spectrophotometry (Shimadzu, Columbia, MD, USA).

## 3. Results and Discussion

Primarily in situ doped polyaniline with dinonylnaphthalene sulfonic acid (DNNSA) exhibits a promising processability in organic solvents. The bulky dopant, DNNSA makes a space between polyaniline molecules so that the polymer molecules slide past each other. However, the bulky nature of DNNSA limits transport of electrical charge, resulting in a low electrical conductivity. Thermal treatment of the primarily doped polyaniline film was observed to increase the electrical conductivity. In this paper, the electrical conductivity and optical properties of the primary doped polyaniline (PANI-DNNSA) are discussed identifying the impacts of functional groups of secondary dopants and doping methods.

**Figure 2:**
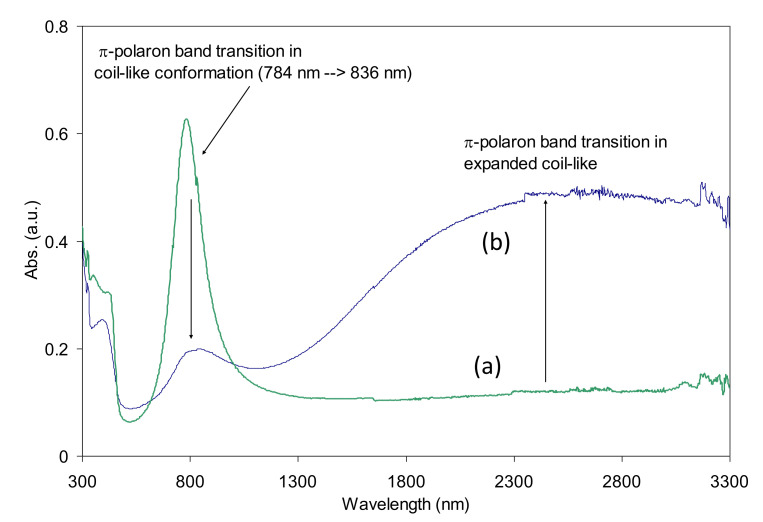
UV–Vis–NIR spectra of PANI-DNNSA films that were air dried (green line, **a**) and thermally treated (blue line, **b**), respectively.

**Figure 3:**
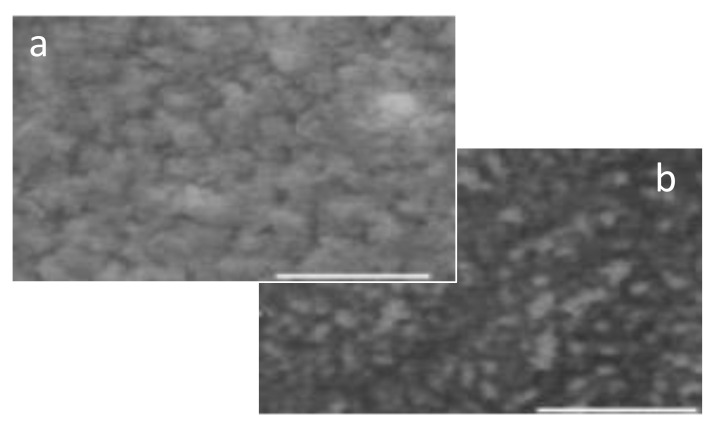
SEM image of PANI-DNNSA films that were: (**a**) air-dried (bar scale: 10 um) and (**b**) thermally treated (bar scale: 100 um).

**Primary in Situ Doped PANI-DNNSA.** The absorption peak of the primarily doped PANI-DNNSA film observed at around 782 nm was assigned to the polaron band in coil-like conformation of polyaniline which was found to be decreased when the films were thermally treated as shown in Figure 2. The effect of thermal treatment was also observed in the NIR region, where the polaron bands of expanded coil-like conformation were observed to be increased. In Figure 3, the SEM image shows the bulky characteristics of the primarily doped PANI-DNNSA film that was air-dried. The bulky nature was observed to decrease upon thermal treatment. The changes in the film morphology support the results of characterization of the optical property in Figure 2.

**Figure 4:**
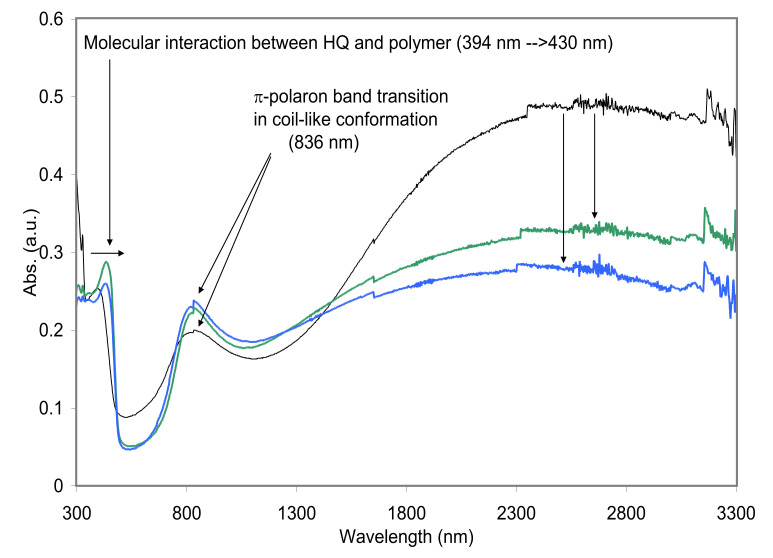
UV–Vis–NIR spectra of PANI-DNNSA films (black) that were film doped with HQ (green and blue). The sample with blue was dipped in BCS for 30 s additionally.

**Secondary Film Doping.** As a typical secondary dopant, hydroquinone (HQ) was applied to the primarily doped polyaniline (PANI-DNNSA), where the interaction between polyaniline and HQ was detected in UV-Vis-NIR absorption profiles in Figure 4, where π-π* absorption of HQ post-(film) doped polyaniline was observed, shifting the absorption peak from 394 to 430 nm. (Film doping conditions: (green curve in Figure 4) 5 *w/v*% HQ in BCS, for 30 s at room temperature and (blue curve in Figure 4) dipped in BCS for 30 s additionally) The red shift (ca. 36 nm) of the absorption peak indicates post-dopant HQ molecule-induced delocalization of π*-electrons along polyaniline molecule. However, the absorption of polaron band at NIR region was measured to be decreased showing no significant molecular packing effect.

**Figure 5:**
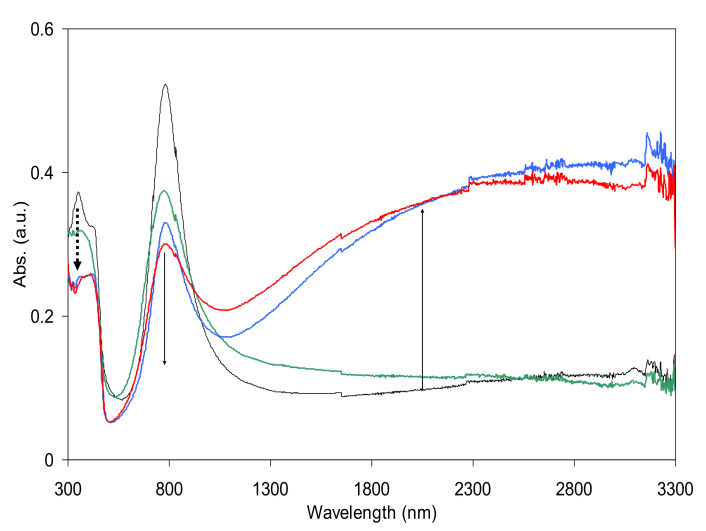
UV–Vis–NIR spectra of PANI-DNNSA films that were film doped with TSAm. The films were air-dried (black line), thermally treated after (green), before (blue), or before and after (red) doping with TSAm. Thermal treatment condition: 150 °C, 30 min in oven.

TSAm is another class of secondary dopants that have sulfonyl groups on it. The sulfonyl functional groups of TSAm plays an important role to increase the electrical conductivity of primarily doped polyaniline, inducing a closely packed molecular conformation. The dopant size of TSAm may be small enough to penetrate through the bulky primarily doped polyaniline molecules that were previously formed by bulky primary dopants, DNNSA via in situ doping process. Figure 5 shows the absorption curves of TSAm doped PANI-DNNSA film. (Film doping condition: (black curve in Figure 5) air dried after film doping, thermal treated after (green), thermal treated before (blue), or thermal treated before and after (red) film doping. TSAm post (film) doping did not show any significant doping effect (black and green curves in Figure 5). The NIR absorption profile after thermal treated in primarily doped PANI-DNNSA film was observed to maintain after the secondary doping was conducted with TSAm. The resultant conductivity of in situ doped polyaniline film was found to be 0.025 S/cm, while it is 2.27 S/cm for film doped film without post thermal treatment.

N-ethyl-*p*-toluenesulfoneamide (E-TSAm) and N-hydroxybenzenesulfoneamide (HBSAm) were applied using in situ and post film doping methods, for which the interaction effects of ethyl and hydroxyl groups of the dopants toward polyaniline molecules. The π-polaron band from coil-like conformation of the secondary doped samples in absorption spectra was found to stay even after the secondary doping process. Surface resistance of the film doped polyaniline in HBSAm/BCS and E-TSAm/BCS solutions (5 *w/v*% each) was measured to be 3220 Ω/sq and 3050 Ω/sq, respectively. The resultant conductivity of the E-TSAm film-doped PAC 1003 was found to be 12.56 S/cm using the two-probe conductivity method and AFM-thickness measurement.

PTSA was found to have significant effect in enhancing electrical conductivity of polyaniline films, where the dopant was used with various solvents [7]. The addition of small amounts of TSAm to PTSA doping solution was reported to increase the electrical conductivity further, where the TSAm was known to have some plasticizing effect, maintaining the conductivity by PTSA. Interestingly, the conductivities observed for secondary doped polyaniline films in PTSA/BCS (5 *w/v*%) and PTSA/TSAm/BCS (5/0.5 *w/v*%) solutions were found to be 334 and 165 S/cm using the four-probe method (Au electrode) as shown in Table 1. UV-Vis-NIR absorptions of the secondary film-doped polyaniline films in PTSA/BCS (5 *w/v*%) and PTSA/TSAm/BCS (5/0.5 *w/v*%) solutions were observed to have substantial absorption profile starting from 500 nm throughout the NIR spectral range, showing strong absorption band of free charge carrier tail. The strong absorption profile explains that the secondary dopants contribute to have extended coil like confirmation for the polyaniline molecules, showing enhanced polaron band.

DBSA is a well-known dopant for polyaniline, incurring good electrical conductivity and thermal stability. Film-doped polyaniline in DBSA/BCS (5 *w/v*%) solution was found to have a two-probe electrical conductivity of 30.7 S/cm. Absorption studies showed that DBSA-doped polyaniline films had better extended coil-like conformation than that of in situ doped films.

**Secondary in Situ (Solution) Doping.** In the case of in situ (solution) HQ doping of the primary doped polyaniline (PANI-DNNSA), the absorption intensity in the NIR range was observed to be decreased. The conductivity of the HQ-doped PANI-DNNSA films was found to be 0.03 S/cm after in situ doping. However, after additional film doping steps were applied on the in situ doped polyaniline film, the conductivity was increased to 1.30 S/cm.

In situ doping of BP and HBSA at a molar ratio of 1:0.3 with PANI-DNNSA was found to provide a poor quality of resulting polyaniline film on glass substrate and the in situ doped polyaniline solution was found to form a gelation in a container. The gelation may happen when bifunctional group of dopants (BP and HBSA) attracts two polyaniline molecules. By reducing the doping level from 1:0.3 to a 1:0.1 molar ratio, the gelation problem was reduced, and the polymer film quality was observed to be improved. However, the resultant surface resistances of the secondary doped PANI-DNNSA films were found to have 0.58 MΩ/sq for BP and 5.72 MΩ/sq for HBSA.

As a result of in situ doping polyaniline with a TSAm doping solution, the absorption band was observed to be changed significantly in the NIR spectral region. Interestingly, after post-thermal treatment of the secondary doped PANI-DNNSA, a synergistic effect was observed. The absorption of polaron band (peak at 870 nm, coil-like conformation of polyaniline) was found to be red-shifted along with further increases in the absorption intensity in NIR band. It shows that coil-like polymer molecules are extended having a closer packing with each other. Gel formation of the in situ TSAm doped polyaniline solution was found 3 days after the doping process was carried out. The gelation of in situ TSAm doped solution was partially decreased by diluting the polyaniline and dopant (TSAm) solutions with xylene and BCS, respectively. However, gelation in the in situ dopant–polymer solution happened again between 7~10 days after the doping-polymer solution was used. Application of optimized doping level and elucidation of the gelation mechanism seem to be critical for aging the solution further.

In the case of in situ doping, the molar ratio of polyaniline and dopant (E-TSAm and HBSAm) was adjusted to 1:0.3. In situ doped secondary doped polyaniline films that were heat-treated after spin-coating were found to have a limited electrical conductivity showing surface resistance of more than 2 MΩ/sq.

Secondary in situ doped polyaniline films that were doped in PTSA/BCS (5 *w/v%*) were found to be non-conductive showing 2-probe surface resistance of ca. 10 MΩ, which was similar to the results discussed earlier in the section on TSAm-based solution doping. The secondary in situ doped polyaniline film showed a significant absorption band at around 800 nm, which explained the solution doped polyaniline films maintained a coil-like conformation of primarily doped polyaniline.

SDP-TSAm (9:1 molar ratio) and DBSA in situ doped polyaniline films were found to have surface resistance of 1069 Ω/sq and 13.35 MΩ/sq, respectively.

Overall, the approach of in situ (solution) doping for PANI-DNNSA solution was not effective to increase electrical conductivity with gelation being the issue during aging.

**Secondary Vapor Doping.** Due to the low molecular weight of thymol and carvacrol, secondary doping may be achieved by a vapor phase doping process. In addition to this, these dopants were reported to show lower toxicity compared to *m*-cresol. The secondary dopants, thymol has isopropyl group on phenol in the two-positions and carvacrol has the groups in two- and five-positions. According to the functional groups, the thermal properties are changed, for which thymol has melting temperature of 50 °C, while 2 °C for carvacrol. The boiling points of thymol and carvacrol are reported to be ca. 230 ˚C. In order to create a vapor in a beaker, the temperature of a hotplate was set to 150 and 100 °C for thymol and carvacrol, respectively. If the heating temperature is set too high, there could be harmful action destroying target polyaniline films. The electrical conductivities of the two doped polyaniline films are listed in the Table 1, 25.2 S/cm for thymol after 30 min doping and 48.5 S/cm for carvacrol. Optimization of the vapor doping condition will increase the electrical conductivity to metallic conductivity. In the case of thymol, extending doping time was found to increase absorption in NIR spectra and electrical conductivity of the secondary doped PANI-DNNSA films. The vapor doping process may work as follows: (1) Penetration of a vapor of the dopants throughout nano-porous PANI-DNNSA film; (2) Washing out excessive and unbound primary bulky dopants, DNNSA, and impure substances with quenched vapor on polyaniline film; (3) Induction of extended coil-like conformation of polyaniline in the film along with secondary dopants; and (4) Formation of fully extended conformation of polyaniline molecules by heat treatment. Table 1 shows the electrical conductivity of the secondary doped PANI-DNNSA films, where the secondary doped polyaniline with PTSA shows one of the best electrical conductivity results, which was 334 S/cm.

## 4. Conclusions

This work shows that functional groups of the secondary dopants and the doping methods provide high impacts on modulating optical and electrical properties of the primarily doped PANI-DNNSA film. The secondary doping was processed using solution, film and vapor doping methods. Among the three doping methods, film doping was identified as the best option to increase electrical conductivity of PANI-DNNSA film. The resultant electrical conductivity was 334 S/cm, increasing the electrical conductivity 2000 times. Optimization of the doping methods is expected to increase the electrical conductivity even further.

## Figures and Tables

**Table 1:** Electrical Conductivity of Chemically Doped Polyaniline films.

Secondary Dopants/ Doping Methods	Thickness (nm)	Resistivity (Ω.cm)	Surface Resistance (Ω/sq)	Conductivity (S/cm) ^1^
N/A (Primary, in situ)	438	6.085	138,853	0.16
Thymol/Vapor	232	0.04	1712	25.16
Carvacrol/Vapor	200	0.021	1032	48.51
PTSA/Film	209	0.003	144	333.92
PTSA/TSAm/Film	200	0.006	190	165.22

^1^ measured using 4-probe conductivity after thermal treatment.

## Data Availability

The data presented in this study are available on request from the corresponding author.
